# Bark Thermal Insulation Panels: An Explorative Study on the Effects of Bark Species

**DOI:** 10.3390/polym12092140

**Published:** 2020-09-19

**Authors:** Günther Kain, Eugenia Mariana Tudor, Marius-Catalin Barbu

**Affiliations:** 1 Forest Products Technology and Timber Construction Department, Salzburg University of Applied Sciences, Markt 136a, 5431 Kuchl, Austria; gkain.lba@fh-salzburg.ac.at (G.K.); eugenia.tudor@fh-salzburg.ac.at (E.M.T.); 2 Higher Technical College Hallstatt, Lahnstraße 69, 4830 Hallstatt, Austria; 3 Faculty for Wood Engineering, Transilvania University of Brasov, B-dul. Eroilor nr. 29, 500036 Brasov, Romania

**Keywords:** tree bark, thermal insulation panels, thermal conductivity, green building materials

## Abstract

Tree bark is a byproduct of the timber industry which accrues in large amounts, because approximately 10% of the volume a log is bark. Bark is used primarily for low-value applications such as fuel or as a soil covering material in agriculture. Within the present study, thermal insulation panels made from larch, pine, spruce, fir and oak tree bark with different resins (urea formaldehyde, melamine formaldehyde, Quebracho, Mimosa) as a binder are discussed. Also, the properties of panels made from larch bark mixed with industrial popcorn are investigated. The physical-mechanical properties of the panels, which are dependent on panel density, bark species, resin type, resin content and particle size, are analyzed. The bark species has a minor effect on the mechanical characteristics of the panels, while the compression ratio is important for the panel strength, and hence, barks with lower bulk density are preferable. Under laboratory conditions, panels made with green tannin resins proved to have adequate properties for practical use. The addition of popcorn is a means to lower the panel density, but the water absorption of such panels is comparably high. The bark type has a minor effect on the thermal conductivity of the panels; rather, this parameter is predominantly affected by the panel density.

## 1. Introduction

Tree bark is the protective layer of a tree. It defends a tree’s vascular cambium from mechanical damage, frost, heat, fires and fungi attack [1,2], and also provides partial structural support [3]. Bark consists of secondary phloem, periderm and nonconductive rhytidome [4]. Its properties vary strongly between species according to the tree’s ecological strategy [5]. Differences exist with regard to thickness, stiffness, water content and density. The risk of forest fires and stem size was shown to explain a large part of the variation of bark thickness on a global scale [6]. The morphology of bark is influenced by other functions too, such as photosynthesis [7], water retention and the storage of nonstructural carbohydrates [1]. Bark functionalities are partly conflictive, i.e., the bark tissue has to prevent the excessive loss of water on the surface of a tree and also should enable the exchange of CO_2_ and O_2_ for photosynthesis and respiration [8].

Analyses of the natural functions of bark suggest a potential for using this natural material as a green thermal insulation material. Early studies showed that the average thermal conductivity of bark is 20% lower than that of wood [9]. A more recent investigation suggested using tree bark as a technical insulation material because of its favorable internal structure, flame retardant properties, low density and fungicidal properties [10].

These potentials were investigated by producing light bark insulation panels out of *Pinaceae* bark with a density of less than 500 kg/m³. The thermal conductivity of the panels was determined to be at least 0.05 W/(m*K), and the mechanical properties were shown to be adequate for use as an insulation material [11]. The thermal diffusivity of such panels is much lower than with standard insulation materials, favoring the prevention of overheating or quick cooling [12]. The panels were prepared using urea formaldehyde and a tannin-hexamine resin as a binder. The latter was shown to have a great potential for green bark insulation panels [13].

Bark panels have also been proposed as decorative wall claddings, and were presented in Austria’s contribution to the Solar Decathlon 2013 in the US (L.I.S.I. House) [14].

Tree bark is a highly heterogeneous material, consisting of various material phases which hinder its technical workability. Nevertheless, the multiphase character of bark displays potential for panel optimization [15], due to characteristics such as steered particle orientation [16].

Bark panels have been discussed in literature extensively e.g., [11,12,13]. Nevertheless, the effect of different bark species on the characteristics of bark insulation panels has not been investigated to date. This is of great importance, because different tree species are processed in different areas of Europe and the world [17,18]. From an ecological point of view, it is important to use building materials which are locally or regionally available to avoid significant CO_2_ emissions due to transport [19]. Therefore, it is important to determine whether, for example, pine species (available in central Europe and Scandinavia) can be used for bark insulation panels in the same way as oak bark (available in Eastern Europe) [20]. 

The aim of this study is to investigate the mechanical, physical and thermal properties of composite panels made of five bark species bonded with conventional adhesives (urea-formaldehyde, melamine-formaldehyde) and green adhesives based on Mimosa and Quebracho tannins, and the influence of bark species, density and particle size on the tested panels. Finally, the question of whether bark can be combined with low density popcorn for the manufacture of low-density recyclable insulation panels was addressed.

## 2. Materials and Methods 

Bark chips (*Picea abies*, *Abies alba*, *Larix decidua*, *Pinus sylvestris*, *Quercus* spp.) were sourced from small sawmills in Salzburg and Upper Austria. The chips were collected at several spots at an approximate depth of 30 cm to prevent untypical boundary effects [21]. A vacuum dryer (Brunner High VAC-S/HV-S1, Hannover, Germany) was used to dry the chips from an initial moisture content (MC) of approximately 100% to 6%. The dry particles were shredded using an R40 four-shaft-shredder (Untha Shredding Technology, Kuchl, Austria). Within the shredder, a sieve was installed to obtain the following particles: 6 > x1 < 10, 8 < x2 < 13, 13 < x3 < 30, 10 < x4 < 45 mm.

Some larch bark particles were mixed with 50 wt-% (based on the dry mass) pre-expanded industrial corn (Balanceboard, Pfleiderer, Neumarkt, Germany) in order to lower the density.

Urea formaldehyde (UF) resin (Prefere 10F102) from Metadynea (Krems, Austria) was used as a standard resin in the present investigation. A melamine formaldehyde (MUF) resin (Prefere 10H927) from Metadynea was also used. Tannin extracts from Mimosa (Acacia mearnsii, Phenotan AG, Tanac S.A., Montenegro, Brazil) and quebracho (*Schinopsis balancae* spp., Colatan GT 10, Markmann GmbH, Hamburg, Germany) were used for the green resin. The tannin resin was prepared by mixing 50 wt-% tannin extract powder and 50 wt-% water in a mechanical stirrer. The pH of the mixture was raised to 9 using a 32% NaOH solution. Finally, 8% hexamethylenetetramine (≥99%, Merck Schuchardt OHG, Hohenbrunn, Germany) was added as a hardener. The resin amount (solid content), as shown in Table 1 and Table 2, was calculated based on the dry mass of the bark and was mixed with the particles in a plough share mixer.

Insulation panels from varying bark species, density, resin type, resin content and particle size were manufactured in a laboratory hydraulic heated press. An overview of the experimental design can be found in Table 1 (for spruce, oak, spruce/fir and pine) and Table 2 (for larch and larch mixed with popcorn). In the current explorative study, due to the high number of variables, a full factorial design was not applied. This limited the detection of small effects; as such, the current study is mainly focused on the primary effects.

**Figure 1:**
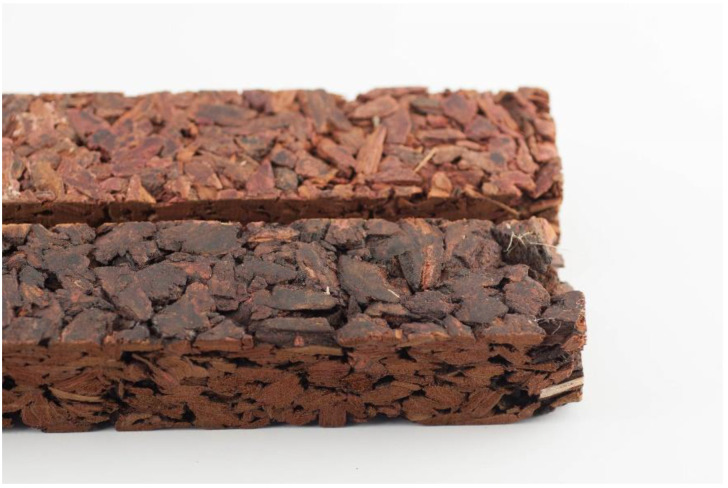
Larch bark insulation panel (glued with tannin in the foreground and UF in the background (thickness = 20 mm, density = 400 kg/m³).

Panels with a size of 350 × 240 × 20 mm³ (Figure 1) were pressed in a Höfer HLOP 280 (Taiskirchen, Austria) laboratory press at a plate temperature of 180 °C and a press factor of 20 s/mm (i.e., significantly higher than in an industrial application). After pressing, the panels were stored in a climate room (20 °C/65% relative humidity (RH)) until weight constancy, and test specimens were cut according to EN 326-1 [22].

Characterization of the panels was conducted by measuring their modulus of rupture (MOR—EN 310 [23]) and internal bond (IB—EN 319 [24]) using a mechanical testing machine (Zwick Roell Z 250). Thickness swelling (TS) and water absorption (WA) after 24 h of water immersion (TS—EN 317 [25]) were determined as well. The panels’ thermal conductivities (TC—EN 12667 [26]) were measured using a EP500 lambda-meter, manufactured by the Lambda Measurement Technologies Corporation (Cincinnati, OH, USA), at an average temperature of 10 °C and a temperature difference between the measurement plates of 15 K.

The results of the physical-mechanical testing were analyzed using a multivariate ANOVA. The panel density was considered as a covariate in the statistical model. The explanatory power is exclusively attributable to each of the individual explanatory variables and was evaluated using partial eta-squared values [27].

**Figure 2:**
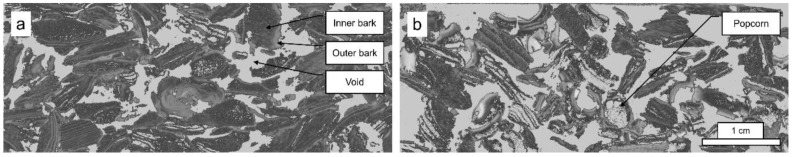
CT-tomograms, resolution 30 µm; (**a**) larch bark insulation panel, 417 kg/m³, thickness 20 mm; (**b**) larch bark + popcorn (50 wt-%), 257 kg/m³, thickness 20 mm.

The structures of the bark boards of two specimens were analyzed by X-ray computed tomography using a Nanotom 180 NF submicro CT device (GE Measurement & Control, Rotterdam, The Netherlands) with a flat panel detector (2304 × 2304 pixels), applying a voltage of 60 kV and a measurement current of 410 µA. Samples with a size of 50 × 50 × 30 mm³ were scanned at a resolution of 30 microns (Figure 2).

## 3. Results and Discussion

The samples’ MC after conditioning (20 °C/65% RH) varied between 12.2 and 15.6%. The equilibrium moisture content of bark samples at 20 °C/65% RH was determined to be up to 3% higher than that of wood, which is in good agreement with the findings of Standke and Schneider [28], who reported that MC variations in bark are twice as high as those in wood, and with Niemz [29], who reported that the equilibrium moisture content of bark is slightly higher than that of the corresponding wood species.

The results of the ANOVA are summarized in Table 3, showing which factors statistically significantly influenced the panel properties. The statistical model for the dependent variables (IB, MOR, MOE, TS, WA, TC) was statistically highly significant (*p* < 0.001) for all variables and the explanatory power of the model was high, as shown by partial η^2^-values higher than 0.83 for all investigated panel properties.

### 3.1. Internal Bond

**Figure 3:**
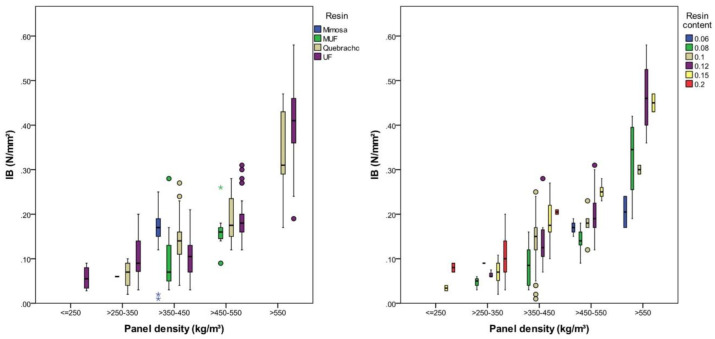
IB according to panel density, resin type and resin content.

The internal bond (IB) of the investigated panels is highly significantly (*p* < 0.001), being affected by the panel density, bark type, resin type, resin content and particle size. The explanatory power (shown by partial eta-squared values) is highest with density (0.50), resin content (0.36) and resin type (0.16) (Table 2). The IB ranged between 0.06 (SD = 0.03) N/mm² for panels with a density between 200 and 250 kg/m³ and 0.36 (SD = 0.11) N/mm² with a density between 550 and 600 kg/m³. On average, the IB increased by 0.1 N/mm² (*p* < 0.001) per 100 kg/m³ increase in density. Focusing on panels with a density between 450 and 550 kg/m³, the IB increased by 0.009 N/mm² with increasing the resin content by 1% (Figure 3).

For the other density classes, the coherence was similar (Table 4). Panels bound with Quebracho tannin and UF resin had an IB superior to that of panels glued with MUF resin. Mimosa tannin was only evaluated for a density of 400 kg/m³, leading to an IB that was 40% higher than that of panels using Quebracho tannin as the adhesive (Table 4), which is in accordance with a study comparing different flavonoid extracts as resin systems for bark particleboards [13].

### 3.2. Moduli of Rupture and Elasticity

**Figure 4:**
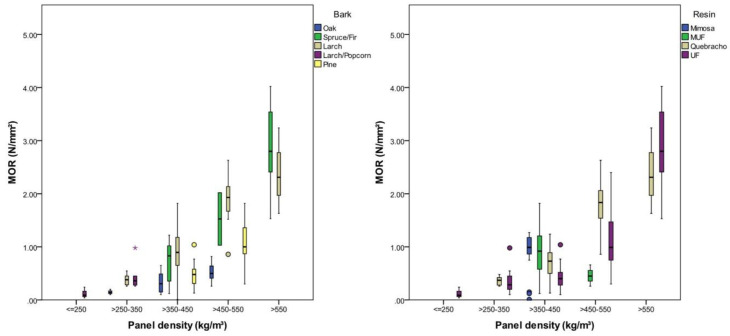
MOR of the investigated panels according to panel density, bark type and resin type.

The modulus of rupture (MOR) is highly significantly (*p* < 0.001), being affected by panel density, bark type and resin type. It was found that 58% of the variation in the MOR could be attributed to density differences, 43% was caused by different bark material and 14% by different resins. The resin content did not show a significant effect on the MOR in this investigation. The MOR was lowest for panels with a density between 200 and 250 kg/m³ with 0.12 (SD = 0.1) N/mm² and highest for panels with a density between 550 and 600 kg/m³ with 2.76 (SD = 0.81) N/mm². On average, the MOR increased by 0.7 N/mm² (*p* < 0.001) with a density increase of 100 kg/m³. Panels with a comparable density between 350 and 450 kg/m³ had an average MOR of 0.33 (SD = 0.19) N/mm² with oak, 0.48 (SD = 0.22) N/mm² with pine, 0.70 (SD = 0.39) N/mm² with spruce/fir and 0.88 (SD = 0.37) N/mm² with larch bark (Table 5). The resin type had a low but significant effect on the MOR in this study, showing the best results with MUF and Mimosa tannin at comparable densities (Figure 4).

**Figure 5:**
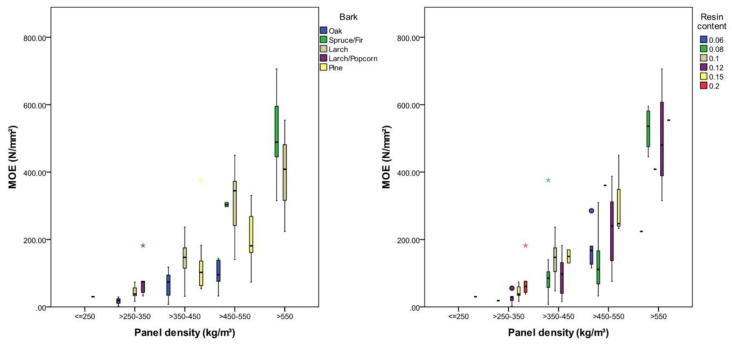
MOE according to panel density, bark type and resin content.

The modulus of elasticity (MOE) was highly significantly (*p* < 0.001), being affected by density, bark type, and resin content, with magnitude of effect in descending order (Table 6); the higher the density and the resin content, the higher the MOE. A minimum MOE of 30 N/mm² was observed with panels with a density between 200 and 250 kg/m³, and a maximum MOE of 484 (SD = 125) N/mm² was observed with a density between 550 and 600 kg/m³. As an average of all investigated panels, the MOE was shown to increase by 140 N/mm² per increase of 100 kg/m³ in panel density. The lowest MOE values were observed with oak bark and the highest with larch bark (Figure 5). Panels with a density between 450 and 550 kg/m³ showed an average MOE of 98 N/mm² (SD = 36 N/mm²) with oak bark and 312 kg/mm² (SD = 91 N/mm²) with larch bark. A high resin content did not clearly improve the MOE. The resin content only explains 16% of the variation in the MOE; its effect thereon seems to be strongly influenced by other factors, and no clear explanation could be derived in this study (Table 6).

It was shown that bark particle board are weaker than panels with wood particles. Whilst low-density wood particleboard (10% isocyanate resin) with a density between 250 and 500 kg/m³ had an IB between 0.2 and 0.6 N/mm², a MOR between 2.5 and 15 N/mm² and a MOE between 1000 and 2500 N/mm² [30], bark particleboard with the same density range (6 to 20% different resins) showed an IB between 0.09 and 0.18 N/mm², a MOR ranging from 0.36 to 1.08 N/mm² and an MOE between 49 and 197 N/mm². The lower mechanical properties of bark particleboard can be explained by the low cellulose content of bark [31], and by thin, brittle phellogen layers, separating the particles [32]. Another reason for the low mechanical strength of bark particleboard is that bark is a porous material which absorbs resin, and therefore, adhesion levels between particles are low [33].

The mechanical properties of the present softwood bark particleboards were comparable to those of other low-density particleboards (100–500 kg/m³) produced from renewable resources (e.g., kenaf core, bagasse), reporting MOR-values between 0 and 7 N/mm² and IB-values between 0.02 and 0.17 N/mm² [34,35].

Measurements showed that the physical-mechanical properties (MOR, IB, and TS 24 h) of the bark-based panels had a competitive edge with commonly available insulation boards [11]. The stability of the panels (expressed by IB) exceeded that of most standard insulation materials, but the panels were also significantly heavier.

The density of the investigated panels was positively correlated with IB, MOR and MOE, which is consistent with other composites, whose density is higher than that of the bulk material [34]. IB and MOE could be improved by incorporating a higher resin content; this principle was less clear when focusing on low densities. A possible explanation for this is the low compaction when the final board density is insignificantly higher than the bulk density of the particles [36]. The resin type showed a significant influence on IB and MOR, but the reasons for this were unclear, indicating that other parameters like particle wetting, suck up of the resin or particle contact were the limiting factors in production. The bark type significantly affected MOR and MOE. It was shown that panels made from low density barks (larch, pine, larch+popcorn) had a higher MOR and MOE than panels made from oak and spruce/fir bark at the same density. This is a result of the higher compression ratio when using low density particles. The IB was insignificantly affected (*η*^2^ = 0.08) by bark species.

Coarse-grained particles also positively influenced the MOR and MOE of wood particleboard [37,38], an effect which could not be confirmed for bark particleboard in the current study, indicating that the MOR and MOE of low density bark panels are limited by the lack of particle contact due to low compression ratios. These properties are depicted in Figure 5.

### 3.3. Thickness Swelling and Water Absorption

**Figure 6:**
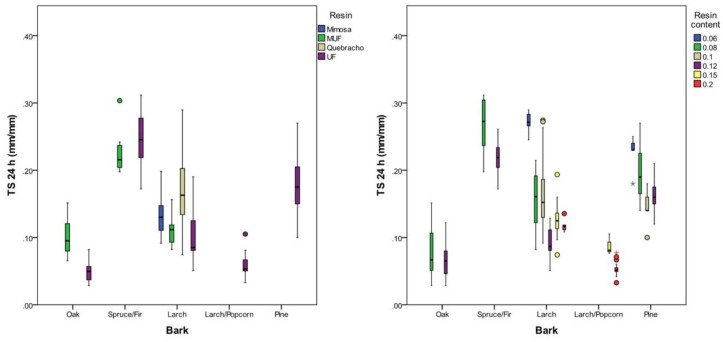
TS after 24 h of water immersion according to bark type, resin content and type.

The bark type, resin type and resin content highly significantly (*p* < 0.001) influenced the thickness swelling (TS) of the panels. It was found that 64% of the variations in the TS could be explained by the bark type, and 31% and 26% by the resin content and type, respectively. TS was lowest with oak bark, i.e., approximately 10%, and was significantly higher for the other bark types (10–30%). Higher resin content proved to result in lower TS for all bark species. MUF with 11 (SD = 2) % and UF resin with 10 (SD = 4)% proved to result in the lowest TS when focusing on larch bark panels (Table 7). Panels made with Mimosa and Quebracho tannin-based resin showed a TS of 13 (SD = 2) and 17 (SD = 5) %, respectively, and are disadvantageous in this respect (Figure 6).

TS after 24 h of water storage was limited in a study focusing on insulation materials made from reed mace, with 15% [39]. It was shown that, for the bark boards, a higher resin content (>10%) was necessary to reach this benchmark. This is consistent with the findings of Kim et al. [40]. Higher density results in higher TS, as is known from the literature [41], but this could not be confirmed in the present study, probably due to low compression. The use of oak bark led to a lower TS due to the lower compaction because of the bark’s higher density (which nonetheless had an adverse effect on the mechanical properties). TS could be further reduced by the addition of wax additives, water repellents or alternative resin systems like pMDI. 

**Figure 7:**
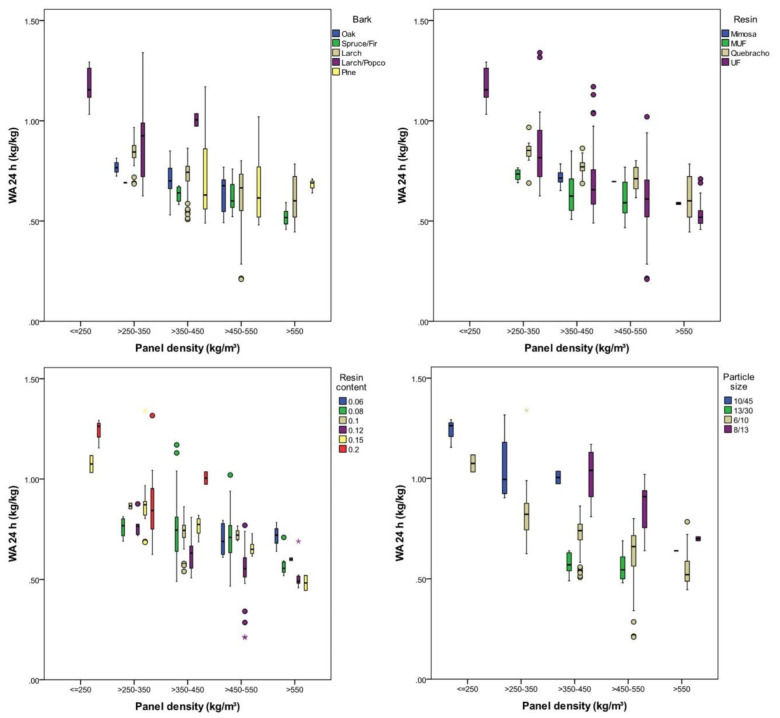
WA after 24 h of water immersion according to density, bark type, resin content, and particle size.

Water absorption (WA) was highly significantly (*p* < 0.001) affected by the panel density, bark type, resin type, resin content and particle size. The explanatory power for the variation in WA was highest with density (47%) and particle size (48%) and lowest with the resin type (15%); the higher the density, the lower the WA, ranging from 117 (SD = 11) % with the lightest panels (200–250 kg/m³) to 56 (SD = 9) % with the heaviest panels (550–600 kg/m³) (Figure 7). 

The WA was the only characteristic investigated which was significantly affected by particle size. It was higher when the particles were smaller, which was a result of the higher specific surface areas of smaller particles. As a result, more water was absorbed by the particles. This was not the case for the larch/popcorn mixture (Table 8). Panels made from coarse-grained particles (x3 and x4) showed a WA of 62% on average, whilst the panels with fine-grained particles (x1 and x2) took up 73% on average. Focusing on panels with a density between 450 and 550 kg/m³ (apart from 20%, all resin contents were used), a higher resin content resulted in lower WA, with lowest being 66 (SD = 4) % using 15% resin content. A probable reason for this is that an abundance of resin sealed the particle surfaces, preventing the absorption of water. Finally, with regard to bark species, a WA between 0.63 and 0.73 was observed with oak, spruce/fir, larch and pine and 100% with the larch/popcorn mixture (Figure 7). 

### 3.4. Thermal Conductivity

**Figure 8:**
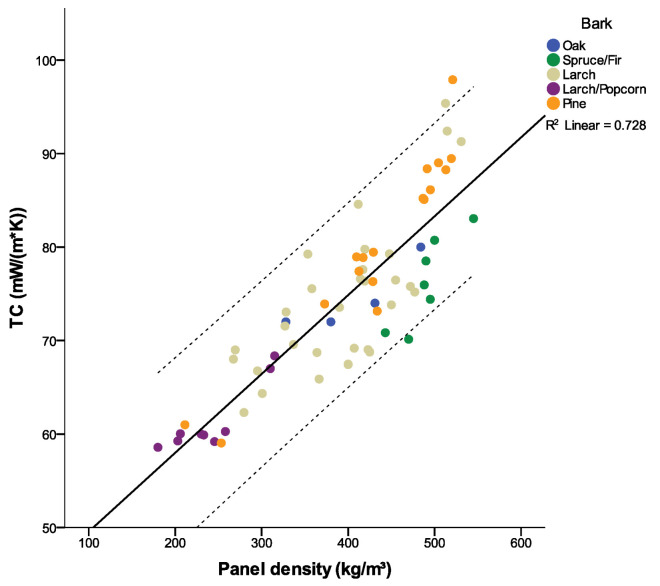
TC according to panel density with 95% confidence interval.

The thermal conductivity (TC) of the investigated panels was highly significantly (*p* < 0.001) affected by panel density. It was found that 67% of the variation in the TC could be explained by the varying density. The lowest TC was measured with 59 mW/(m*K) and a density of 180 kg/m³ (Larch+Popcorn). The TC increased by 8.4 mW/(mK) by increasing the panel density by 100 kg/m³ (Figure 8). This is in accordance with studies on other insulation materials showing that low density of insulation panels reduces their TC due to a high void content in the composite [42]. Small pores are advantageous in this respect, because the air in such voids is static, and heat convection has a minor effect. Therefore, the panel structure offers significant optimization potential [16,38].

The bark type significantly (*p* < 0.1) affected the TC, but only explained 13% of the TC variation (Table 9), and the differences in TC caused by different bark type were small (Table 9). Regression analyses between panel density and TC calculated for each bark type yielded significant (*p* < 0.02) models for all bark types except for oak bark (*p* = 0.11). Referring the TC of all panels to a density of 400 kg/m² using the regression models, the TC of spruce/fir (0.064 W/(mK)) was lowest, followed by larch/popcorn (0.072 W/(mK)), oak (0.074 W/(mK)), larch (0.075 W/(mK)) and pine (0.077 W/(mK)).

Focusing on the thermal characteristics, the bark-based panels showed a minimum thermal conductivity value of 0.059 W/(m*K), which is higher than those of very light insulation boards (e.g., mineral wool, polystyrene with approximately 0.03 W/(m*K)). This disadvantage is compensated for by the low thermal diffusivity of bark [12]. This makes the material especially suitable for use as insulation layers which need to prevent quick cooling or overheating in summer. The bark type only had a minor effect on the TC of the panels; this variable was predominantly affected by the panel density, in accordance with Brombacher et al. [42], who made the same observation for wood fiber panels and various combined materials. From this point of view, barks with a lower density (specific gravity *Pinus sylvestris* 0.40, *Quercus* spp. 0.59) did not contribute to a lower TC.

The potential to lower the density of the bark panels in order to increase porosity (53% with a density of 200 kg/m³ [43]) is low, due to panel stability. The compression ratio (board density divided by particle density) of boards with 220 kg/m³ was only 0.6, whilst the lower limit for compression ratio yielding sufficiently stable panels was estimated to be around 0.7 for low-density particleboards [35]. Significant potential to increase porosity without lowering mechanical stability could be nonetheless exploited by using targeted particle size mixtures [44] and possibly expandable or more reactive resins. Another strategy for the optimization of TC, as shown in another study, is the targeted orientation of particles. If they are oriented predominantly parallel to the panel plane, the global TC could be reduced by 13% [16]. 

Another strategy to increase the compression ratio, and consequently, the mechanical properties, is the use of a low-density resource basis. This would reduce the panel’s TC as well. Expandable fillers (expandable polystyrene granulates) have already been successfully applied to lower the density of particleboard [45]. The present study shows that the addition of popcorn lowers the panels’ density from a minimum of approximately 250 kg/m³ with larch bark to a minimum of 200 kg/m³. On the other hand, the WA of these panels was very high (around 100%), which lowers their potential for real life applications.

## 4. Conclusions

The presented bark insulation panels proved to be an adequate material for special purpose insulation where very low thermal conductivity is not the primary focus. This is the case if, e.g., the heat storage capacity is suitable for installing a thermal mass in a building [46]. Bark insulation panels could be an interesting material for the fabrication of insulation layers which are under mechanical stress due to their favorable strength properties [47].

All investigated bark species (spruce, fir, pine, larch and oak) were shown to be suitable for insulation panel production. At the same density, panels from barks with a low bulk density (pine, larch) are advantageous because their compression ratios are higher, which improves the mechanical characteristics. The addition of low-density aggregates like popcorn lowers the density but, at the same time, lowers moisture resistance, leading to a high WA; as such, this is not recommended.

Green tannin resins showed very good performance, i.e., comparable with their synthetic counterparts. It is to be considered that the tannin resins were applied under laboratory conditions, and that problems might occur in an industrial setting due to the rapid change in viscosity that occurs directly after preparation, which might negatively influence their use [13].

## Figures and Tables

**Table 1:** Experimental design for bark insulation panels (spruce, oak, spruce/fir and pine).

Bark	Resin Type	Resin Content	Target Density (kg/m³)	Particle Size	Number of Specimens
Spruce	Loose bulk		258		
Oak	UF	8	300	6/10	1
			350		1
			400		1
			450		1
		12	300		1
			350		1
			400		1
			450		1
	MUF	8	350		1
			400		1
			450		1
		12	350		1
			400		1
			450		1
Spruce/Fir	UF	8	400	6/10	1
			450		2
		12	400		2
			450		2
Pine	Loose bulk	0	200	8/13	1
			250	8/13	1
	UF	6	500	13/30	1
		8	400	13/30	3
				8/13	3
			500	13/30	2
				8/13	2
		10	400	13/30	1
		12	350	13/30	1
			400	13/30	3
			500	13/30	2
				8/13	3

**Table 2:** Experimental design for bark insulation panels (larch and larch/popcorn).

Bark	Resin Type	Resin Content	Target Density (kg/m³)	Particle Size	Number of Specimens
Larch	UF	8	400		1
			450		1
		12	300		1
			350		1
			400		2
			450		1
		15	250		1
			300		1
	MUF	8	400		2
			450		1
	Tannin-Quebracho	6	500		2
		8	400		2
		10	350		2
			400		7
			500		2
		15	250		2
			300		2
			350		2
			400		2
			500		2
		20	250		2
	Tannin-Mimosa	10	400		7
Larch/Popcorn	UF	15	250	6/10	1
		20	200	10/45	1
			250	10/45	1
			300	6/10	1
			300	10/45	2
			350	6/10	2
			350	10/45	3

**Table 3:** Results of the ANOVA with *p*-values and η²-values for the explanatory variables.

	IB		MOR		MOE		TS		WA		TC	
	*p*	*η* ^2^	*p*	*η* ^2^	*p*	*η* ^2^	*p*	*η* ^2^	*p*	*η* ^2^	*p*	*η* ^2^
Model	0.000	0.873	0.000	0.832	0.000	0.883	0.000	0.835	0.000	0.845	0.000	0.904
Density	0.000	0.496	0.000	0.582	0.000	0.688	0.100	0.022	0.000	0.471	0.000	0.666
Bark	0.000	0.083	0.000	0.426	0.000	0.252	0.000	0.643	0.000	0.234	0.095	0.128
Resin	0.000	0.161	0.000	0.143	0.080	0.049	0.000	0.256	0.000	0.157	0.285	0.078
Resin content	0.000	0.360	0.101	0.048	0.000	0.157	0.000	0.314	0.000	0.325	0.271	0.126
Particle size	0.000	0.074	0.342	0.012	0.368	0.015	0.238	0.010	0.000	0.475	0.922	0.000

**Table 4:** IB of panels with varying resin types and contents.

IB (N/mm²)																
Resin	Resin c.	Panel Density (kg/m³)														
		≤250.00			>250–350			>350–450			>450–550			>550		
		M	S	N	M	S	N	M	S	N	M	S	N	M	S	N
Mimosa	0.10							0.17	0.04	52						
MUF	0.08				0.06	-	1	0.06	0.03	4	0.14	0.03	4			
	0.12				0.06	-	2	0.17	0.11	3	0.20	0.05	3			
Queb.	0.06										0.16	0.01	4	0.17	-	1
	0.08							0.14	0.01	3	0.14	-	2			
	0.10				0.09	-	1	0.12	0.04	60	0.18	0.04	5	0.30	-	2
	0.15				0.06	0.03	8	0.18	0.05	12	0.25	0.02	5	0.45	-	2
	0.20				0.07	0.03	4									
UF	0.06										0.18	0.01	4	0.24	-	1
	0.08				0.04	-	2	0.08	0.04	15	0.15	0.02	10	0.33	0.10	4
	0.10							0.11	0.03	4						
	0.12				0.08	-	1	0.12	0.02	5	0.20	0.05	24	0.46	0.08	8
	0.15	0.03	-	2	0.08	0.02	3									
	0.20	0.08	-	2	0.12	0.05	13	0.21	-	2						

**Table 5:** MOR dependent on bark and resin type.

		MOR (N/mm²)														
Bark	Resin	Panel Density (kg/m³)														
		≤250			>250–350			>350–450			>450–550			>550		
		M	S	N	M	S	N	M	S	N	M	S	N	M	S	N
Oak	MUF							0.45	0.18	4	0.46	0.13	8			
	UF				0.15	0.04	4	0.29	0.18	10	0.58	0.18	9			
Spruce/Fir	MUF							0.70	0.39	19						
	UF										1.53	-	2	2.85	0.82	13
Larch	Mimosa							0.91	0.35	23						
	MUF							1.15	0.32	20						
	Quebracho				0.36	0.08	6	0.68	0.29	25	1.79	0.59	6	2.39	0.81	3
	UF				0.40	0.13	5	0.42	-	2	2.02	0.33	5			
Larch/Popcorn	UF	0.12	0.10	3	0.47	0.30	5									
Pine	UF							0.48	0.22	21	1.07	0.37	19			

**Table 6:** MOE dependent on bark and resin content.

		MOE (N/mm²)														
Bark	Resin c.	Panel Density (kg/m³)														
		≤250			>250–350			>350–450			>450–550			>550		
		M	S	N	M	S	N	M	S	N	M	S	N	M	S	N
Oak	0.08				18.58	-	1	70.07	34.83	12	78.31	35.9	9			
	0.12				15.79	19.21	5	63.50	39.90	7	115.29	37.63	9			
Spruce/Fir	0.08										309.68	-	1	528.18	66.89	6
	0.12										297.62	-	1	494.01	128.69	9
Larch	0.06										285.26	-	1	223.82	-	1
	0.08							139.91	-	1	140.31	-	1			
	0.10							149.84	46.46	45	360.54	-	1	408.38	-	1
	0.12				34.94	18.80	3	35.63	6.38	25	342.68	61.64	5			
	0.15				47.07	20.99	6	149.70	-	2	309.73	121.78	3	553.94	-	1
	0.20				41.94	-	2									
Larch/Popcorn	0.15				32.38	-	1									
	0.20	30.00	-	1	94.00	60.64	18									
Pine	0.06										151.35	28.54	25			
	0.08							111.02	96.28	54	168.76	65.40	36			
	0.10							89.55	40.93	29						
	0.12							139.38	30.59	20	258.68	64.47	71			

**Table 7:** TS after 24 h of water soaking.

		TS 24 h (mm/mm)											
		Resin Type											
Bark	Resin c.	UF			MUF			Quebracho			Mimosa		
		M	S	N	M	S	N	M	S	N	M	S	N
Oak	0.08	0.05	0.01	16	0.11	0.02	9						
	0.12	0.05	0.01	12	0.08	0.02	9						
Spruce/Fir	0.08	0.29	0.02	13	0.23	0.04	16						
	012	0.22	0.03	18	0.22	0.02	18						
Larch	0.06							0.27	0.02	13			
	0.08	0.16	0.03	5	0.12	0.03	19	0.20	0.02	13			
	0.10	0.10						0.18	0.04	192	0.13	0.02	183
	0.12	0.09	0.02	15	0.10	0.01	18						
	0.15	0.10		2				0.13	0.02	65			
	0.20							0.12	0.01	13			
Larch/Popcorn	0.15	0.09	0.02	3									
	0.20	0.05	0.01	28									
Pine	0.06	0.23	0.03	30									
	0.08	0.19	0.04	95									
	0.10	0.14	0.03	29									
	0.12	0.16	0.02	95									

**Table 8:** WA after 24 h of water soaking.

			WA 24 h (%)														
			Panel Density (kg/m³)														
Bark	Resin	Resin c.	≤250			>250–350			>350–450			>450–550			>550		
			M	S	N	M	S	N	M	S	N	M	S	N	M	S	N
Oak	MUF	0.08				0.74	-	1	0.81	0.05	4	0.73	0.04	4			
		0.12				0.75	-	2	0.68	0.03	3	0.69	0.01	4			
	UF	0.08				0.80	-	2	0.70	0.07	6	0.65	0.11	4			
		0.12							0.64	0.08	4	0.54	0.02	5			
Spruce/Fir	MUF	0.08							0.68	0.01	3	0.60	-	1	0.59	-	2
		0.12										0.56	0.03	6			
	UF	0.08										0.71	0.03	6	0.54	0.02	4
		0.12							0.60	-	2	0.60	0.03	4	0.49	0.02	8
Larch	MUF	0.08							0.56	0.03	3	0.51	0.04	3			
		0.12							0.54	0.03	6						
	Queb.	0.06													0.78	-	1
		0.08										0.82	-	1			
		0.10							0.78	-	2	0.61	-	1			
		0.15				0.78	-	2	0.73	-	1	0.61	-	2			
		0.20				0.86	-	1									
	UF	0.08							0.67	-	1	0.60	-	2			
		0.12				0.79	0.08	3	0.65	0.02	5	0.26	0.06	4			
		0.15				0.80	-	2									
Larch/Popcorn	UF	0.15	1.07		2	1.34	-	1									
		0.20	1.24	0.07	3	0.88	0.19	13	1.00	-	2						
Pine	UF	0.06										0.64	0.04	4	0.64	-	1
		0.08							0.80	0.26	9	0.94	0.05	5	0.71	-	1
		0.10							0.56	0.02	5						
		0.12							0.81	-	1	0.57	0.09	13	0.69	-	1

**Table 9:** TC according to density class and bark type.

	TC (mW/(m*K))											
	Panel Density (kg/m³)											
	≤250			>250–350			>350–450			>450–550		
Bark	M	S	N	M	S	N	M	S	N	M	S	N
Oak				72.00		1	73.00	-	2	80.00	-	1
Spruce/Fir							70.84	-	1	77.13	4.64	6
Larch				67.60	4.60	5	69.85	2.85	7	75.49	-	2
Larch/Popcorn	59.49	0.58	6	65.21	4.33	3						
Pine	60.99	-	1	59.04	-	1	76.87	2.52	7	88.68	4.10	8
